# A Reusable Capillary Flow-Driven Microfluidic System for Abscisic Acid Detection Using a Competitive Immunoassay

**DOI:** 10.3390/s25020411

**Published:** 2025-01-12

**Authors:** Cristiana Domingues, Marta S. C. Rodrigues, Pedro G. M. Condelipes, Ana Margarida Fortes, Virginia Chu, João Pedro Conde

**Affiliations:** 1 Instituto de Engenharia de Sistemas e Computadores–Microsistemas e Nanotecnologias (INESC-MN), Rua Alves Redol, 1000-029 Lisbon, Portugal; cdomingues@inesc-mn.pt (C.D.); marta.rodrigues@tecnico.ulisboa.pt (M.S.C.R.); pedromonteiro@biosurfit.com (P.G.M.C.); vchu@inesc-mn.pt (V.C.); 2 Instituto de Biossistemas e Ciências Integrativas (BioISI), Faculdade de Ciências de Lisboa, Universidade de Lisboa, 1749-016 Lisbon, Portugal; amfortes@ciencias.ulisboa.pt; 3 Department of Bioengineering, Instituto Superior Técnico, Avenida Rovisco Pais, 1049-001 Lisbon, Portugal

**Keywords:** microfluidics, abscisic acid, capillarity, hydrophilic polydimethylsiloxane copolymer (PDMS-PEG), portability

## Abstract

Point-of-care (PoC) devices offer a promising solution for fast, portable, and easy-to-use diagnostics. These characteristics are particularly relevant in agrifood fields like viticulture where the early detection of plant stresses is crucial to crop yield. Microfluidics, with its low reagent volume requirements, is well-suited for such applications. Self-driven microfluidic devices, which rely on capillary forces for fluid motion, offer an attractive alternative by eliminating the need for external pumps and complex fluid control systems. However, traditional microfluidic prototyping materials like polydimethylsiloxane (PDMS) present challenges due to their hydrophobic nature. This paper presents the development of a reusable, portable, capillary-driven microfluidic platform based on a PDMS-PEG (polyethylene glycol) copolymer designed for the rapid low-cost detection of abscisic acid (ABA), a key biomarker for the onset of ripening of non-climacteric fruits and drought stress in vines. By employing passive fluid transport mechanisms, such as capillary-driven sequential flow, this platform enables precise biological and chemical screenings while maintaining portability and ease of use. A simplified field-ready sample processing method is used to prepare the grapes for analysis.

## 1. Introduction

Molecular biomarkers are crucial for monitoring the health of plants, animals, and humans, aiding in disease diagnosis and early intervention. To prevent the spread of infections, early detection is the key. This was shown in the COVID-19 pandemic, where rapid tests were essential [1,2,3]. This is also the case in agriculture, where early detection can prevent the spread of infections, optimize crop health, and enhance resource management [4,5]. However, access to traditional diagnostic tools is often limited by high costs, bulky laboratory equipment, and the need for specialized personnel. Besides this, the turnaround time for receiving the results of the analysis is often long, delaying the start of the intervention. This highlights the importance of developing rapid, portable, affordable, integrated, and easy-to-use PoC systems for molecular detection that are accessible even in resource-limited settings that could enable rapid in-field diagnostics where timely decisions are essential for crop health and yield optimization [6,7,8,9,10].

This is particularly important for the early detection of plant stresses, which helps prevent disease spread, reduce resource waste, and improve overall crop management for sustainable agriculture. Abscisic acid—ABA (Molar Mass: 264.32 g/mol)—is a plant hormone that is involved in the plant’s response to physical environment changes and stresses, such as temperature changes, dehydration, and seed germination. Its levels rise under stress and during the ripening of non-climacteric fruits, like grapes, particularly in the *veraison* stage when the levels of anthocyanins increase and cause the berries to change colour [11]. ABA concentrations vary by grapevine cultivar, so what is normal for one genotype may indicate stress in another. Monitoring ABA over time helps establish a baseline for assessing plant health. Typically, ABA concentrations range from 10^−5^ to 10^−1^ mg/mL in grape berries [6,11,12]. High-Performance Liquid Chromatography (HPLC) is widely employed for detecting ABA, as it effectively separates and identifies components within a solution [13]. Despite its accuracy and reliability, HPLC is impractical for field applications due to its large cumbersome equipment, the need for trained operators, lengthy analysis times, and significant operational costs [12,14].

Lateral Flow Assays (LFAs) are a widely recognized PoC technology, known for their simplicity and use in applications such as the detection of COVID-19 infections or pregnancy rapid tests. In these devices, the solution flows along a porous material (typically nitrocellulose) strip with immobilized reagents, where the reagents move laterally, producing visible results when detecting a target substance. Although LFAs are easy to use, affordable, and deliver quick results, they offer limited control over fluid flow, lack integration with advanced sensors, and are typically single-use. In contrast, channel-based capillary microfluidic devices provide more advanced capabilities by utilizing capillary forces to control fluid flow through patterned channels, allowing for complex assays with sequential flows and sensor integration. These systems offer greater precision and reusability, with no cross-contamination between uses, and are ideal for applications requiring higher sensitivity, such as biomarker monitoring or advanced diagnostics in agriculture and personalized medicine [15]. Despite the potential advantages of POC methods for detecting ABA in grapevines, the availability of such technologies remains limited, and most current detection methods still rely on traditional laboratory-based approaches. For instance, optical aptamer-based sensors with microfluidic interfaces have been reported for detecting plant hormones like ABA, offering sensitive and rapid detection through optical signals. However, they do not present an explicit sample treatment capable of being used in the field [14].

Microfluidic devices, particularly self-driven systems using capillary forces, process small volumes of samples and reagents with high precision, enabling the fast and sensitive detection of plant biomarkers and environmental factors, overcoming the challenge of needing external equipment for fluid control [8,16,17,18,19]. Microfluidic systems designed for sequential reagent flow are highly efficient tools for conducting complex biochemical assays, including immunoassays. These systems use precisely engineered channels and capillary forces to control the movement of various reagents in a set order, allowing for multiple steps in the assay to occur automatically. One key feature of these microfluidic platforms is their ability to handle intermediate and final washing steps, which are critical for removing unbound reagents and ensuring the accuracy and sensitivity of the assay. The system can introduce wash solutions between different reagent flows without user intervention, enhancing consistency and minimizing the risk of contamination [15]. PDMS, commonly used in microfluidics, is limited by its hydrophobic nature (contact angle ~110°), making it unsuitable for capillary-driven flow [20,21]. To improve its wettability, methods like surface modifications, including oxygen plasma treatments and hydrophilic coatings, have been developed to make PDMS more effective for capillary microfluidic applications [15,22,23,24,25].

However, the effectiveness of these surface modification techniques should be assessed based on factors such as processing time, procedural complexity, required equipment, and their compatibility with downstream application processes. A balance must be achieved between enhancing PDMS’s hydrophilicity and ensuring that the modifications are feasible, scalable, and compatible with the practical needs of agricultural PoC diagnostics. By addressing these challenges, microfluidic devices can offer a scalable, portable solution for monitoring plant stress, disease, and other factors affecting crop health and productivity [26,27]. PDMS-PEG is a hybrid material that combines the flexibility and hydrophobicity of polydimethylsiloxane with the hydrophilicity and biocompatibility of polyethylene glycol, making it ideal for antifouling, biomedical, and microfluidic applications [15].

With the goal of detecting stresses in grapevines in the field with minimal user intervention, in this study, a competitive immunoassay for the detection of ABA using a capillary-driven microfluidic device was developed based on the acquisition of a fluorescence signal. Key steps included developing a bead packing method and adjusting the volumes and flow rates of the assay, as well as introducing intermediate washing steps, for a successful operation of the capillary chip. A simplified sampling cleaning protocol so that the whole analysis process can be performed in the field with no need for bulky laboratory equipment was also developed. Additionally, in this work, the device was fully characterized in terms of its reusability and the method used to restore it to its original state after multiple uses.

## 2. Materials and Methods

### 2.1. Fabrication of the Hard Masks and the SU-8 Moulds for the Microfluidic Devices—Externally Pumped and Capillary

The microfluidic designs were created using Computer Assisted Design (CAD) software (AutoCAD 2024 (version 24.3), Autodesk, San Francisco, CA, USA). An aluminum hard mask on Corning^®^ Eagle glass was microfabricated using direct-write laser lithography (Heidelberg DWLII, Heidelberg Instruments, Heidelberg, Germany) and subsequent aluminum wet etching (Gravure Aluminium Etchant, Microchemicals, Ulm, Germany). Two aluminum masks were fabricated to allow for the patterning of two channel areas with different heights. These were used in conjunction with a negative photoresist, SU-8 2015 (Microchem, Newton, MA, USA) for 20 μm or 50 µm layers and SU-8 50 (Microchem, Newton, MA, USA) for 100 μm layers to create a two-level mould pattern. The SU-8 photoresists were subjected to UV light (UV KUB-2, KLOÉ, Saint-Mathieu-de-Tréviers, France) exposure through the corresponding aluminum hard mask, followed by a post-exposure baking process. The development was then carried out using propylene glycol methyl ether acetate (PGMEA, 99.5%, Sigma-Aldrich, St. Louis, MO, USA) to remove the unexposed photoresist. The SU-8 mould was then rinsed with isopropanol and dried with compressed air, followed by a hard bake at 150 °C for 15 min [12].

### 2.2. PMDS Device Fabrication

#### 2.2.1. Standard PDMS

**Figure 1:**
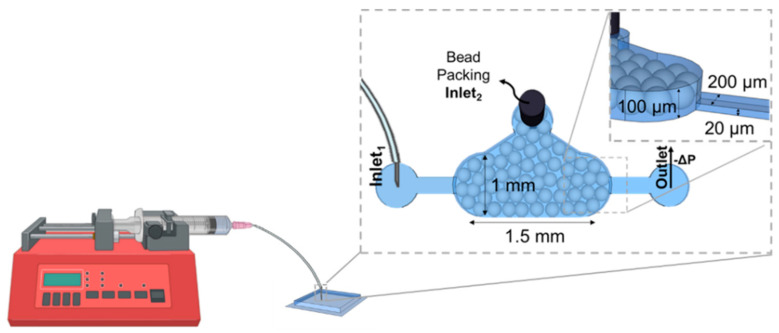
Externally pumped microfluidic device: a 100 µm high channel was utilized for bead packing, while a 20 µm high channel was designated for sample and reagent injection. The lower-height inlet and outlet channels effectively trap microbeads with diameters exceeding 20 µm.

To prepare standard PDMS, a curing agent and elastomer base were mixed in a 1:10 (*w*/*w*) ratio and then degassed for 45 min. Subsequently, the PDMS mixture was poured over the SU-8 mould. To ensure uniformity in the size of all replica moulds, a flat poly (methyl methacrylate) (PMMA, Trinseo S.A., Berwyn, IL, USA) plate was positioned on top to close the device. The assembly was then cured at 70 °C for 90 min. The microfluidic devices were sealed with a 500 μm thick PDMS membrane (fabricated on a silicon wafer under identical curing conditions) by exposing both surfaces to oxygen plasma (Harrick Plasma, Ithaca, NY, USA). To ensure a stable contact angle, the PDMS structures were stored for a minimum of 24 h before use (Figure 1) [12].

#### 2.2.2. Modified PDMS

**Figure 2:**
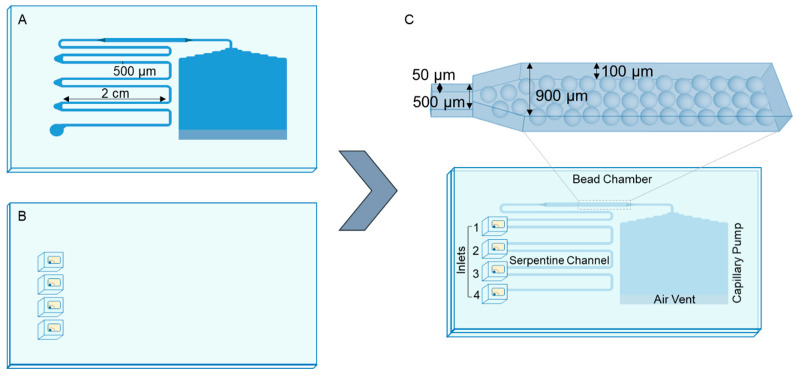
Device assembly, features and operating principles: (**A**) modified PDMS feature layer; (**B**) modified PDMS membrane layer incorporating reservoirs; (**C**) key components of the sealed capillary microfluidic device features: serpentine channel, four reagent entry points (reservoirs), capillary pump, air vent and microbead chamber. The device incorporates dual height levels within the microbead chamber to enable efficient trapping of microbeads. The total fluid volume capacity of the device is approximately 24 µL, with an average flow rate of 1.8 µL/min.

For the modified PDMS, the standard mixture was combined with 1% (*w*/*w*) of dimethylsiloxane-(60–70% ethylene oxide) block copolymer. To fabricate the device’s feature layer, which includes the channels, the detection chamber, and the capillary micropump, 1% copolymer-modified PDMS was poured onto the SU-8 mould. The PDMS mixture was then degassed in a heated desiccator at 40 °C. Once all air bubbles were eliminated, a flat PMMA plate was placed on top to close the device and ensure uniform size across all replicas. The assembled stack was cured for 90 min at 70 °C (Figure 2A). The capillary structures are sealed manually, offering the benefit of easy opening, cleaning, and reuse of the device. To close the channels, a 500 µm membrane is fabricated under identical curing conditions. This membrane was cast from a 6.42 × 3.85 cm, 5 mm thick PMMA frame, with reservoirs engraved and drilled using CNC micromachining (Figure 2B). The reservoirs were constructed with distinct geometries: the upper three were conical in shape, while the fourth featured a rounded-bottom cylindrical configuration. Each reservoir incorporated a 600 μm diameter circular aperture, designed to accommodate metallic adapters for sealing the serpentine channel. The conical geometry was optimized to enhance the efficient transfer of small reagent volumes to the entry points. Additionally, for sealing, both the membrane and capillary structures were fabricated within frames equipped with alignment markers to ensure the accurate positioning of the features relative to the membrane’s entry points (Figure 2C) [15].

Following the fabrication of both modified PDMS layers, the device was assembled using four components: the modified PDMS structure, a matching membrane, and top and bottom PMMA frames embedded with Teflon-coated magnets for sealing. The top frame had openings for the membrane’s reservoirs and a vent at the capillary pump, while the bottom frame ensured proper alignment with a 2 mm depression to securely hold the device [15].

### 2.3. Ultraviolet Ozone (UVO) Treatment

UVO treatment was carried out using a UVO cleaner (1444AX-220, Jelight Company Inc., Irvine, CA, USA). After a significant change in the flow rates, to recover hydrophilicity in the device, the modified PDMS structures were submitted to the treatment, in which both interior surfaces of the device were exposed to a cycle of 5 min of cleaning and 5 min of exhaust time.

### 2.4. Solutions and Chemicals

Polydimethylsiloxane, PDMS, (Sylgard 184) was acquired from Dow Corning Corporation (Midland, MI, USA). Dimethylsiloxane-(60–70% ethylene oxide) block copolymer was acquired from Gelest (Morrisville, PA, USA). An amount of 10 kDa Amicon tubes were obtained from Merck (Alameda Fernão Lopes, Algés, Portugal). Protein A and Q-Sepharose microbeads were purchased from Cytiva (Uppsala, Sweden). Phosphate-Buffer Solution (PBS) 10×, casein (1% *w*/*v*), Alexa Fluor^®^ (A430) NHS ester (succinimidyl ester) from Fisher Scientific (Waltham, MA, USA), anti-ABA polyclonal antibody—AS09 422 (14.3 mg/mL) from Agrisera (Vännäs, Sweden), and ABA [BSA] (4 mg/mL) from Creative Diagnostics (Shirley, NY, USA). ABA (50 mg/mL), DMSO (10 mg/mL), and methanol (MeOH) anhydrous 99.8% were obtained from Sigma-Aldrich (St. Louis, MO, USA).

### 2.5. ABA-BSA Labelling

To prepare the fluorescently labelled ABA-BSA, 20 µL of ABA-BSA was diluted in 480 µL of 0.1 M sodium bicarbonate buffer and then conjugated with 2 µL of Alexa Fluor^®^ (A430) NHS ester, an amine-reactive dye, which had been dissolved in DMSO at a concentration of 10 mg/mL and incubated at room temperature for 60 min in the dark. After incubation, excess dye was removed by washing the mixture in a 10 kDa Amicon tube (Merck, Alameda Fernão Lopes, Algés, Portugal). This was performed by adding 500 µL of PBS at a time, followed by centrifugation at 14,000× *g* for 10 min until the permeate became clear [12].

### 2.6. Protein-A Microbeads Functionalization

The protein-A microbeads (~90 µm) were incubated with an anti-ABA antibody solution by mixing 3 µL of bead stock with 19 µL of anti-ABA antibody at a concentration of 0.2 mg/mL. The incubation lasted 60 min. It is important to ensure that the 3 µL of microbead stock contains a consistent number of microbeads, as any variation in bead quantity across incubations could affect the antibody concentration on the microbeads, thereby influencing the number of available binding sites [12].

#### Microbead Insertion in the Externally Pumped and Capillary Device

To introduce microbeads into the externally pumped microfluidic system, negative pressure was applied at both the outlet and inlet1 using a syringe pump (NE-4000, New Era Pumps, Farmingdale, NY, USA). The channel was first pre-filled with PBS 1× buffer, and a pipette tip containing 20 µL of bead solution was positioned at the bead chamber inlet2, as illustrated in Figure 1. The applied negative pressure induced a flow rate of approximately 6 µL/min, facilitating the packing of microbeads within the detection chamber. Subsequently, the microfluidic system was flushed with PBS 1× at a flow rate of 16 µL/min, and the bead chamber inlet2 was sealed using a 20-gauge metal plug. The bead-packed structure was then immersed in a container filled with deionized (DI) water and refrigerated overnight to remove any air bubbles introduced during the bead-loading process [12].

For the capillary microfluidic device (Figure 2), 3 µL of functionalized microbeads were introduced into the bead chamber before performing the assay. Following the evaporation of the carrier solution, the device was manually sealed and made ready for operation.

### 2.7. ABA Detection Immunoassay

#### 2.7.1. Externally Pumped Device

The bead chamber of the externally pumped device was packed with functionalized microbeads, as described in Section Microbead Insertion in the Externally Pumped and Capillary Device. To minimize non-specific interactions with the PDMS surface of the microchannel and the agarose beads, a 0.1% (*w*/*v*) casein solution was introduced into the system at a flow rate of 1 µL/min for 10 min. Subsequently, the labelled ABA-BSA conjugate (0.03 mg/mL), along with a specified analyte concentration or prepared sample, was delivered into the channel at the same flow rate for 5 min, maintaining a consistent 1% methanol concentration. This process was followed by a PBS 1× washing step at a flow rate of 5 µL/min for 10 min to eliminate unbound ABA-BSA conjugate and analyte [12].

#### 2.7.2. Capillary Device

Following bead insertion into the capillary device, as outlined in Section Microbead Insertion in the Externally Pumped and Capillary Device, the device was manually closed (Figure 2C), reagents were loaded, and the immunoassay was conducted. In this assay, Reservoir 4 was filled with 12 μL of PBS 1×, which was used to prime the device and perform washing steps. Since the assay required only three reagents, Reservoir 3 was loaded with 5 μL of PBS 1×, Reservoir 2 with 4 μL of labelled ABA-BSA conjugate (0.03 mg/mL) mixed with a specified analyte concentration or prepared sample (maintaining a constant methanol concentration of 1%), and Reservoir 1 with 3 μL of blocking agent (0.1% casein).

### 2.8. Grape Collection and Treatment for ABA Detection

To assess the sensitivity of the ABA detection assay, fresh Red Globe table grapes were purchased from a supermarket and stored at −80 °C until use. These mature grapes exhibit naturally low ABA levels in comparison to those harvested at the *veraison* stage [11,12].

**Figure 3:**
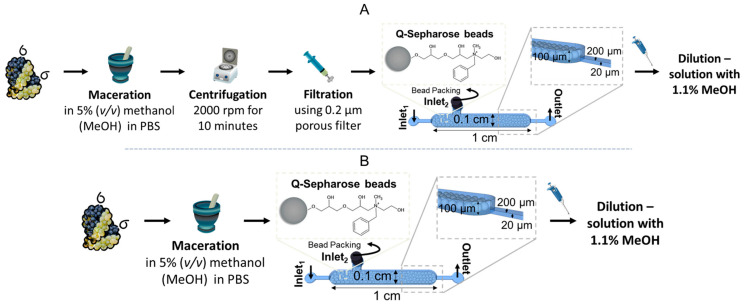
Overview of grape sample treatment steps. (**A**) Comprehensive protocol: The process includes maceration of the sample; centrifugation (2000 rpm, 10 min), filtration through a 0.2 µm pore-sized filter, and a bead-based cleaning step within a microfluidic channel. The microfluidic columns used for cleaning were 1 cm in length, 0.1 cm in width, and 100 µm in height. Additionally, the system featured a smaller channel (200 µm in width and 20 µm in height) specifically designed to trap beads with diameters exceeding 20 µm). (**B**) Simplified protocol: The procedure consists of sample maceration followed directly by a bead-based cleaning step within a microfluidic channel.

To further validate the ABA detection method, a first sample treatment was developed (Figure 3A) [12]. Berries from the Touriga Nacional cultivar were harvested at the *veraison* stage from a local vineyard and categorized based on anthocyanin accumulation into green, intermediate, and dark colour groups. The berries were flash-frozen in liquid nitrogen, transported to the laboratory on dry ice, and stored at −80 °C for subsequent analysis. Sample preparation involved macerating the grape tissue in a mortar using a 5% methanol (MeOH) solution diluted in 1× PBS, at a ratio of 1 g of tissue to 1 mL of solution, for 8 min to facilitate biomarker dissolution and extraction. The resulting mixture was centrifuged at 2000 rpm for 10 min, and the supernatant was collected and filtered through a 0.2 µm Nylon syringe filter. To further eliminate residual contaminants, including debris that passed the initial filtration, the supernatant was purified using externally pumped microfluidic channels packed with Q-Sepharose beads. The sample was passed through the channels at a flow rate of 5 µL/min for 10 min. Finally, all samples were diluted to achieve a consistent 1.1% MeOH composition, as preliminary microfluidic experiments showed that higher MeOH concentrations interfered with the molecular recognition assay.

To load the Q-Sepharose microbeads (~90 µm diameter) into the sample cleaning microfluidic structure (Figure 3), negative pressure was applied at the outlet using a syringe pump. The channel was first filled with PBS 1× buffer, and a pipette tip containing 30 µL of bead solution (prepared by diluting the bead stock 1:10 in 20% ethanol) was positioned at the chamber inlet_2_. The applied negative pressure induced a flow rate of approximately 12 µL/min, facilitating the packing of beads into the sample treatment chamber. Following this, the microfluidic structure was rinsed with PBS 1× at a flow rate of 22 µL/min, and the bead chamber inlet_2_ was sealed using a 20-gauge metal plug.

A centrifuge is not practical for field testing. Therefore, the sample treatment was simplified by eliminating the centrifugation and filtration steps from the original protocol, as shown in Figure 3B. This modified process is fully suitable for field use and does not require specialized personnel to operate the equipment.

### 2.9. Fluorescence Image Acquisition and Data Analysis Using ImageJ

Fluorescence images for the experimental assays were captured using a fluorescence microscope (Leica DMLM, Wetzlar, Germany) equipped with a digital camera (DFC300FX) and a CoolLED lamp (pE-300lite) as the excitation light source. A filter cube with an excitation band-pass of 450–490 nm (blue) and an emission long-pass filter at 515 nm (green) was used. The acquisition parameters were fixed with an exposure time of 2 s, a gain of 1×, and a saturation of 1, all acquired using a 10× objective. The images were then analyzed with ImageJ software 1.49 (National Institutes of Health, Bethesda, MD, USA) [28]. The analysis involved splitting the images into RGB channels, with only the green channel being further analyzed. A region of interest (ROI) was defined around the beads to extract the fluorescence signal. The resulting green channel signal was determined by subtracting the background fluorescence (measured from an equal-sized area outside the bead chamber) from the fluorescence signal inside the ROI. These consistent acquisition settings and analytical methods ensured reliable quantification of the ABA in the micrographs [12].

## 3. Results and Discussion

The goal of this paper is to detect ABA in a capillary-driven microfluidic device. By leveraging the natural capillary forces of the microfluidic system, the platform enables a simple and efficient detection method. ABA, a key biomarker for grape ripening during the *veraison* stage, is detected using a competitive immunoassay. ABA detection will be performed on real grape samples, utilizing a sample treatment method that was previously developed in our group [12]. This sample treatment will be compared to a newly developed field-ready sample treatment introduced in this study.

The user-friendly platform consists of the modified PDMS structure, the membrane layer, and the top and bottom PMMA frames [15]. A master mould was developed, with two distinct heights, to trap the microbeads inside a chamber, as can be seen in Figure 2, and used to carry out the competitive immunoassay. The SU-8 mould for the capillary device was fabricated using photolithography and soft-lithography techniques, as detailed in Section 2.1.

### 3.1. Test of Capillary Device Operation with Food Colouring

The capillary microfluidic system was tested with food dyes, where each different colour can be associated with the reagents required for the immunoassay, to confirm the functionality of the optimized dual-height design in terms of sequential flow.

**Figure 4:**
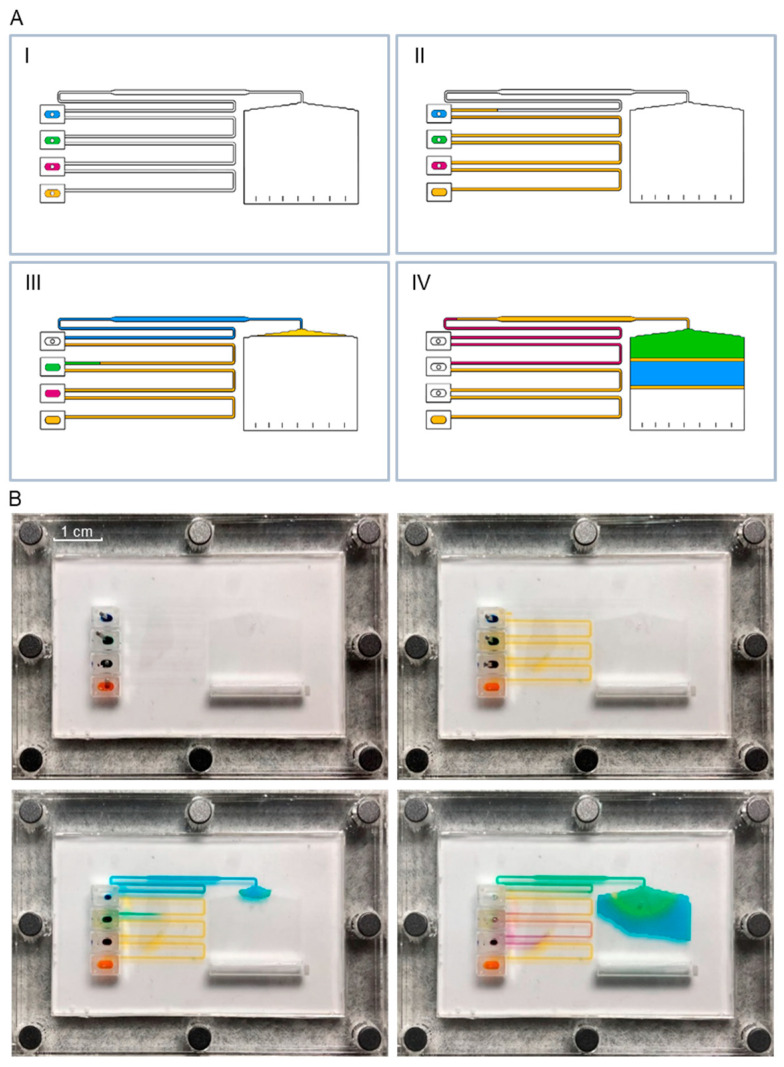
Representation of the device’s operation using food colouring. (**A**) Schematic illustration showing the key steps: reservoir filling (I); device priming - orange (II); and sequential introduction of solutions (blue, green, purple) from the remaining reservoirs (III and IV). (**B**) Photographs demonstrating the proof-of-concept with food colouring. Note that the images are captured from a top-down perspective, which does not reveal the height difference between the channel and the bead chamber.

Device operation starts by sealing the reservoirs with metallic adapters and filling them with the solutions (Section 2.2.2). The volumes used in the proof-of-concept assay were 12, 4, 4, and 4 μL of orange, blue, green, and purple food colouring, respectively. Figure 4 represents the sequential flow of solutions from the moment the reservoirs are filled until the end of the assay. The liquid starts to flow when the bottom metallic plug is pulled from the structure, allowing for the priming of the microchannels. As the priming solution (orange) flows through the remaining three reservoirs, their plugs are sequentially removed, starting from the top to the bottom, allowing for the solutions to enter the device in a controlled manner. The reservoir containing the priming/washing solution facilitates the replication of the intercalated flow of reagents and washes typically utilized in standard immunoassays. The end of the assay is indicated by the filling of the capillary pump.

A correlation can be established between the various colours employed and the reagents in a competitive immunoassay: orange represents the washing buffer, blue represents the blocking agent, green represents the sample (which can be spiked or not spiked with ABA) together with the conjugate, and purple represents the washing buffer. In this assay architecture, the probe antibody to which the target/conjugate will bind must already be present in the device, and, in this case, it is functionalized onto the protein-A microbeads off-chip, as detailed in Section 2.6.

### 3.2. Flow Rate Measurements

The use of liquids with different food colourings to test the performance of the capillary microfluidic device allows for the determination of the position of each reagent at a specific time after flow initiation. In addition to following the sequential flow, the images obtained can be used to calibrate the flow rates between specific points.

To determine the average flow rate of the capillary device, one can use the total assay time and the volume of the microfluidic structure, which is approximately 24 μL. The assay completion time was measured from the beginning of the priming step until the liquid reached the end of the capillary pump. The flow rate in the device stabilizes after the third use, with a completion time of approximately 13 min, resulting in a flow rate of approximately 1.8 μL/min.

**Figure 5:**
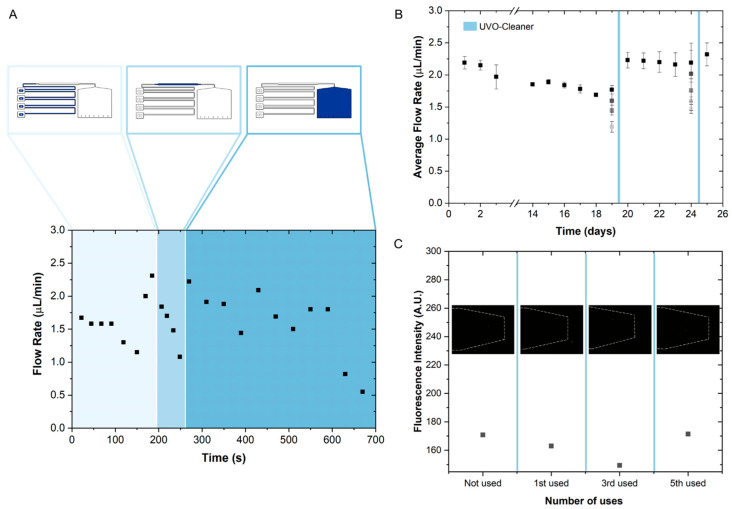
(**A**) Flow rate measurements obtained through image acquisition and calculation at specific time intervals when the liquid is flowing in the serpentine channel, bead chamber (highlighted in blue), and capillary pump. (**B**) Average flow rate measurements by repeated flow of PBS through the microchannels to assess the ability to reuse the device and hydrophilicity recovery after UVO treatment. Each measurement refers to one experiment per day, except for 19 and 24, where several experiments were performed; the first to last are represented from darker to lighter colour, respectively. The error bars represent the standard deviation between two independent devices. (**C**) Fluorescence intensity measurements taken across multiple uses of the same capillary device, with respective acquired fluorescence images. The excitation wavelength was 450–490 nm (blue).

Additionally, the flow rate between specific parts of the device was accessed to determine how the reagents of the immunoassay were flowing through the structure. Several images were acquired throughout the assay, and the area occupied by the liquid was measured, converted to a volume, and divided by the time intervals between the images. Figure 5A shows these results, separated into three parts: the serpentine channel, the bead chamber, and the capillary pump. The obtained flow rate is higher when compared to the one in the ABA detection immunoassay using an externally pumped device, in which reagents flow at 1 µL/min. However, it is lower than the rate used for the final PBS wash of 5 µL/min.

To determine the feasibility of device reuse and possible treatments to recover the hydrophilicity of the membranes, device ageing experiments were performed. To assess the ability to reuse the device, PBS was flowed through the channels and the flow rate was measured each time. The results are expressed as the average flow rate, calculated from the total volume of the structure and the completion time of the assay. The flow rate variations can be seen in Figure 5B. After approximately the three initial applications of the device, the modified PMDS can be reused at least four times, with minimal change in flow rates if used once a day, having a slight decrease at day eighteen. However, if used several times a day, with the first to last represented from darker to lighter colours, respectively, there is a significant decrease in average flow rates. This may occur if the hydrophobic methyl groups in PDMS migrate to the surface, returning to a more stable configuration. Furthermore, the fluid flow accelerates this rearrangement process, especially under repeated flow conditions, which is seen when comparing daily use to multiple uses in a short time.

To recover hydrophilicity in the device, the membranes were exposed to the UV Ozone cleaner for 10 min, as described in Section 2.3. As a result, the surface becomes more hydrophilic because hydroxyl and carbonyl groups are introduced. Figure 5B shows the hydrophilicity recovery of the device, since the flow rate returns approximately to its initial state after the UVO treatments. Just like the first times the device is used, a similar stabilization in the flow rates is seen between days twenty and twenty-three, following UV Ozone treatment. Similar flow rates were also registered after treatment, on days twenty and twenty-five, and on the first use on day one.

An additional study investigated potential fluorescence residues left on the chip after each use of the capillary device. The fluorescence intensity shown in Figure 5C was measured after using and washing the capillary chip. Figure 5C confirms the absence of fluorescence contamination between uses, indicating that the device can be reused successfully, at least five times with no contamination observed. The cleaning protocol between each experiment involves unsealing the device, washing both components, the modified PDMS feature layer (Figure 2A) and modified PDMS membrane layer with reservoirs (Figure 2B), with isopropyl alcohol (IPA) and DI water, and drying them thoroughly with an air gun.

### 3.3. Application of the Platform to a Competitive Immunoassay

**Figure 6:**
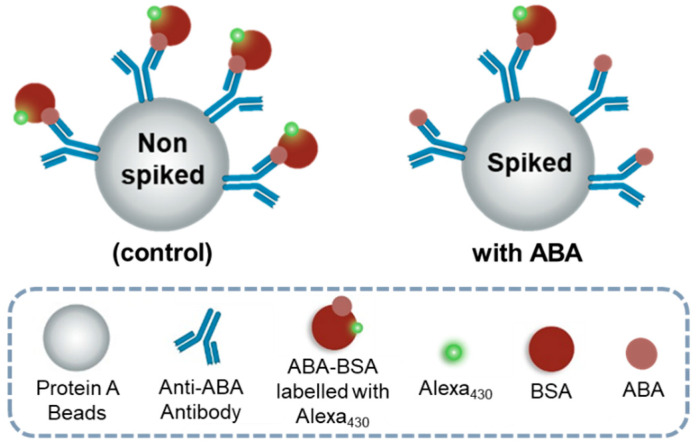
A schematic of the competitive fluorescence immunoassay is presented, comprising three key stages: bead functionalization, in which the anti-ABA antibody is covalently attached to the bead via its constant region, leaving the binding site available for target interaction; a control assay without ABA, where only the labelled ABA-BSA conjugate is present, binding to the anti-ABA antibody and generating the maximum fluorescence signal due to the absence of competition; and an ABA-spiked assay, where both the labelled ABA-BSA conjugate and free ABA analyte compete for antibody binding sites. As the concentration of free ABA increases, the fluorescence signal diminishes due to decreased binding of the labelled conjugate.

A competitive immunoassay was used as a proof-of-concept application in the capillary platform. This assay was developed previously by our work group through immobilizing anti-ABA antibodies on Protein A microbeads using an externally pumped microfluidic channel for optical detection of ABA (Section 2.7.1) [12]. This strategy is schematically summarized in Figure 6. In this setup, the fluorescence signal is maximal in the absence of ABA. However, as ABA concentrations increase, the free analyte competes with the labelled ABA-BSA conjugate for binding to antibody sites, resulting in a reduction in the fluorescence signal. The use of microbeads enhances sensitivity by increasing the surface area for antibody binding and reducing assay time by decreasing diffusion distances. Consistent bead quantities were used to ensure uniform antibody availability and consistent competition conditions across assays. The optimal concentrations of the antibody and conjugate were previously determined to be 0.2 mg/mL for the antibody and 0.03 mg/mL for the conjugate [12].

In the control assay, where no ABA is present, only the labelled ABA-BSA conjugate is introduced into the microchannel. In contrast, for assays involving spiked ABA or treated grape samples, the labelled ABA-BSA conjugate is co-introduced with the sample under analysis.

**Figure 7:**
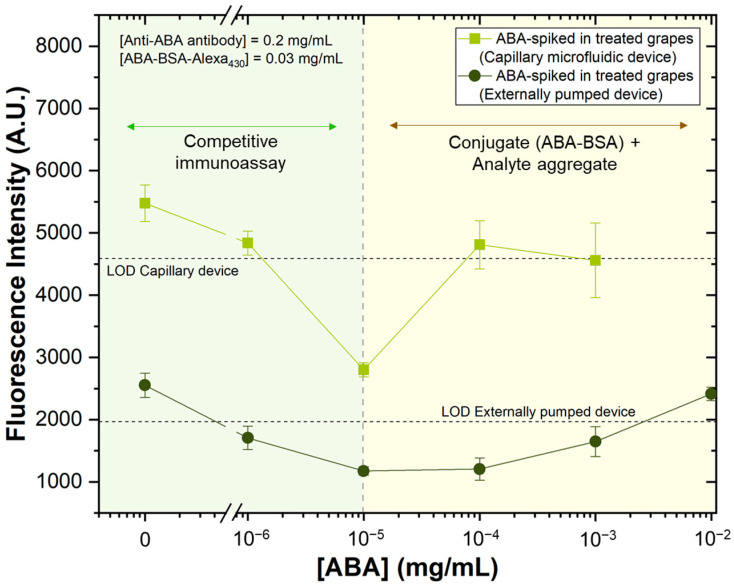
A competitive immunoassay for ABA detection was performed on ABA-spiked treated table grape samples, with ABA concentrations ranging from 0 to 10^−2^ mg/mL (n = 2), using optimized concentrations of anti-ABA antibodies (0.2 mg/mL) and ABA-BSA-Alexa430 (0.03 mg/mL). Fluorescence intensity was measured on two platforms: an externally pumped device and a capillary-driven device. The excitation wavelength was 450–490 nm (blue excitation light). Error bars represent the ± standard deviation.

The relationship between fluorescence signal intensity and ABA concentration in treated table grapes was evaluated, with the results presented in Figure 7. For these experiments, successive dilutions of ABA were spiked into the treated samples to determine the minimum detectable ABA concentration using both the externally pumped device and the capillary device. The sample treatment protocol, detailed schematically in Figure 3A of Section 2.8, including a centrifugation step, was strictly followed.

A competitive immunoassay using an externally pumped device was previously developed [12] and then adapted for the capillary device (Section 2.7.2). Both the flow rate and reagent volumes were adjusted. The standardized flow rate for this capillary device was set to 1.8 μL/min. Figure 7 shows that similar results were obtained with both the externally pumped and capillary devices, with a limit of detection (LOD) from 10^−3^ to 10^−6^ mg/mL and 10^−4^ to 10^−6^ mg/mL, respectively, confirming the applicability of the capillary device for ABA detection. Regarding the assessment of grape ripeness based on fluorescence intensity in on-site applications, it is important to note that grape ripening is accompanied by visible colour changes and an increase in abscisic acid (ABA) concentration, as documented in the literature [11]. These measurements help identify the relevant segment of the curve, enabling accurate quantification of the ABA concentration. For a continuous study of grape ripening, monitoring the same grape bunch over successive seasons would provide valuable insights. This approach would allow for the observation of ABA concentration dynamics over time, offering a clearer picture of the relationship between ABA levels and grape maturation.

The fluorescence response as a function of ABA concentration follows a V-shaped curve. Below 10^−5^ mg/mL, the assay behaves as a typical competitive immunoassay, with fluorescence decreasing as ABA concentration increases. However, at higher concentrations, above 10^−5^ mg/mL, the fluorescence signal increases with rising analyte concentration, suggesting the potential formation of aggregates between the ABA-BSA conjugate and the free analyte in the solution. Therefore, as a part of our method to accurately access the ABA concentration in the sample, a 10× dilution may be used to identify the corresponding segment of the curve and accurately determine the ABA concentration.

#### Sample Treatment in Wine Grapes Samples

**Figure 8:**
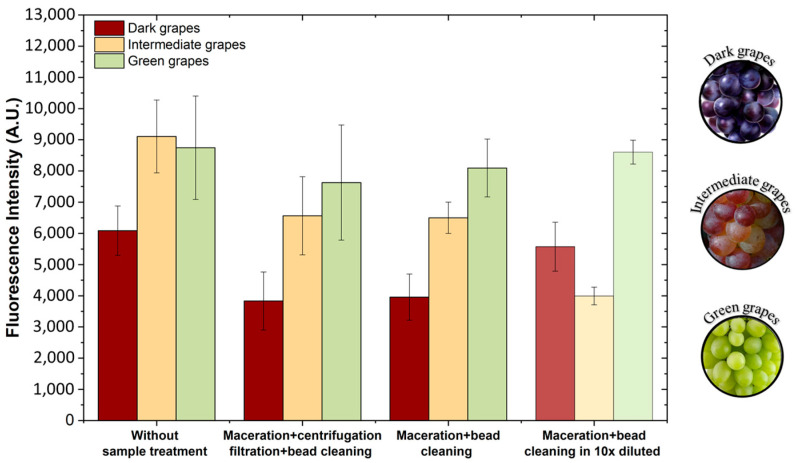
The ABA detection method was validated in real grape samples using the capillary microfluidic device. Three grape samples—dark, intermediate, and green—were selected from a grape cluster at the *veraison* stage. ABA concentrations were measured using the microfluidic competitive immunoassay for ABA detection under three conditions: without sample processing, with sample treatment involving centrifugation, and with simplified treatment that excludes centrifugation and filtration. The final set of bars represents the microfluidic ABA detection competitive immunoassay performed on 10× diluted validation samples subjected to the simplified sample treatment. The excitation wavelength was 450–490 nm (blue excitation light). Error bars represent the ± standard deviation (n = 2).

The grape samples selected to validate the ABA detection method through the capillary device were at the *veraison* stage, the phase where berries undergo colour change. During this stage, ABA levels are known to rise in grapes. In the same grape cluster, berries exhibit varying degrees of ripening, with some remaining green, others turning dark, and a few in between, showing significant ripening heterogeneity. Samples were categorized into three stages—green, intermediate, and dark—based on their visual appearance, as seen in Figure 8**.** “Green” grapes were fully green, “dark” grapes had a clear red colour, while “intermediate” grapes showed both colours, marking the transition. All samples were of the Touriga Nacional cultivar.

The samples used in this study were previously analyzed using HPLC [12]. The ABA concentrations in the intermediate, dark, and green grape samples, measured with HPLC, were approximately 5 × 10^−4^ mg/mL, 4 × 10^−5^ mg/mL, and 0 mg/mL, respectively.

To be able to detect stress biomarkers in grapevines directly in the field—without requiring skilled operators, lengthy analysis times, or high costs—a simplified sample treatment method was developed. This method eliminates the need for equipment typically unavailable in field settings, such as centrifuges (Figure 3B in Section 2.8).

Figure 8 highlights the importance of proper sample pre-treatment. In the first set of results, it is evident that without treatment, it is difficult to distinguish the fluorescence intensity between the green and intermediate samples. In the next set of results, the sample treatment developed in reference [12] was applied, with the microfluidic assay for ABA detection yielding results consistent with those obtained from HPLC analysis. As anticipated in a competitive assay, the absence of ABA in the sample results in a higher fluorescence signal, as observed in the green grape sample. The intermediate and dark grape samples, with ABA concentrations of approximately 10^−4^ mg/mL and 10^−5^ mg/mL, respectively, exhibit fluorescence trends consistent with those presented in Figure 7. In these cases, the fluorescence signal for the 10^−4^ mg/mL ABA sample is greater than that of the 10^−5^ mg/mL sample. However, this pre-treatment requires centrifugation, which is not convenient for use in the field. To address this limitation, a simplified pre-treatment was developed involving only maceration and a bead-cleaning step in a microfluidic channel. The results in Figure 8 show that the fluorescence intensity levels from this simplified treatment were similar to those obtained using the previous method, which includes centrifugation [12]. This demonstrates that sample preparation can be effectively streamlined to just maceration and bead cleaning in a microfluidic channel for use in the field.

In order to address the uncertainty in ABA concentration caused by the V-shaped curve in Figure 7, the processed grape samples previously used for assay validation were diluted by a factor of 10 and reanalysed with the capillary microfluidic system and the simplified sample treatment. The results of these measurements, shown in Figure 8, indicate that after a 10× dilution, the ABA concentrations in the intermediate and dark grape samples are within the ranges of 10^−5^ mg/mL and 10^−6^ mg/mL, respectively. Consequently, the intermediate sample displays a lower fluorescence signal compared to the dark sample. Additionally, the fluorescence signal of the green grape sample remains relatively unchanged after dilution, consistent with expectations, as it closely resembles the signal from the non-ABA-spiked grapes. Therefore, if there is uncertainty regarding the ABA concentration in a sample, further dilutions can help clarify the measurement. However, when performing these measurements, one must take into account that the labelling process (Section 2.5) inherently varies slightly between consecutive runs. These variations result in differences in fluorescence intensity. The key focus should be on observing the overall trend. However, our goal is to determine the concentration of ABA, not just to look at the trend. Observing the trend is simply one step in our method to accurately quantify the ABA concentration. The most accurate approach would be to generate a new calibration curve each time a new labelling is performed. Additionally, it is important to calibrate the ABA concentration using a matrix that closely resembles the sample under study; for example, in the experiments in Figure 8, one should use wine grape matrix.

## 4. Conclusions

A capillary microfluidic biosensing platform for detecting the fruit ripening biomarker abscisic acid (ABA) was successfully developed, demonstrated, and validated. Beyond indicating the onset of fruit ripening, ABA also serves as a biomarker for drought stress, making this platform suitable for the continuous monitoring of ABA levels to assist in managing plant water status. The platform’s functionality was enhanced by incorporating a dimethylsiloxane-(60–70% ethylene oxide) block copolymer into PDMS, rendering it hydrophilic and compatible with capillary-driven applications. The design incorporates a two-height structure to improve microbead trapping within the chamber, while it was demonstrated that the device has the potential to be reused at least four times, enhancing both its environmental sustainability and its user-friendliness.

A sequential introduction of solutions was initially demonstrated using food dyes, followed by a competitive immunoassay for ABA detection. This immunoassay employed fluorescence as the signal transduction method, with anti-ABA antibodies functionalized on the surface of the microbeads trapped within the capillary microfluidic device [12]. Additionally, this study introduced and validated a simplified sample treatment that eliminates the need for centrifugation, thereby enabling the platform’s use for in-field ABA detection.

A key advantage of the proposed device is its magnetic-assisted mechanical sealing, which enables easy opening, cleaning, and reuse. The platform is user-friendly, with three simple steps: reagent loading, device priming, and reservoir opening. With the developed assay, we were able to detect ABA in concentrations ranging from 10^−4^ to 10^−6^ mg/mL using our capillary device, which is within the range of interest [14]. The most used technique, HPLC, has a LOD of around 10^−4^ mg/mL [29]. An alternative microfluidic technique using aptamers as molecular recognition probes was able to obtain a LOD of 10^−5^ mg/mL [14]. This makes our assay as sensitive as the previously reported ones, with the additional advantage that it can be used directly in the field from a raw sample, providing results in just 13 min.

For full automation and portability, future integration of an optical sensor or colorimetric measurements with smartphone readout will be necessary to further enhance the platform’s portability.

## Data Availability

The raw data supporting the conclusions of this article will be made available by the authors on request.
